# Ligand-based virtual screening and inductive learning for identification of SIRT1 inhibitors in natural products

**DOI:** 10.1038/srep19312

**Published:** 2016-01-25

**Authors:** Yunan Sun, Hui Zhou, Hongmei Zhu, Siu-wai Leung

**Affiliations:** 1 State Key Laboratory of Quality Research in Chinese Medicine, Institute of Chinese Medical Sciences, University of Macau, Macao, China; 2 School of Informatics, University of Edinburgh, Edinburgh EH8 9AB, United Kingdom

## Abstract

Sirtuin 1 (SIRT1) is a nicotinamide adenine dinucleotide-dependent deacetylase, and its dysregulation can lead to ageing, diabetes, and cancer. From 346 experimentally confirmed SIRT1 inhibitors, an inhibitor structure pattern was generated by inductive logic programming (ILP) with DMax Chemistry Assistant software. The pattern contained amide, amine, and hetero-aromatic five-membered rings, each of which had a hetero-atom and an unsubstituted atom at a distance of 2. According to this pattern, a ligand-based virtual screening of 1 444 880 active compounds from Chinese herbs identified 12 compounds as inhibitors of SIRT1. Three compounds (ZINC08790006, ZINC08792229, and ZINC08792355) had high affinity (−7.3, −7.8, and −8.6 kcal/mol, respectively) for SIRT1 as estimated by molecular docking software AutoDock Vina. This study demonstrated a use of ILP and background knowledge in machine learning to facilitate virtual screening.

Sirtuin 1 (SIRT1) is a nicotinamide adenine dinucleotide (NAD^+^)-dependent histone deacetylase with an anti-ageing function^1^. Figure 1 shows the catalytic process between SIRT1 and its substrates (some non-histones or histones)^2^,^3^,^4^,^5^. Through this deacetylation process, SIRT1 is involved in various cellular processes including cell proliferation, cellular responses, DNA repair, and cell apoptosis^2^,^3^,^4^,^5^. SIRT1 is a potential therapeutic target for type 2 diabetes and cancer^1^,^6^. Thus, specific SIRT1 ligands with biological activities may help to delineate the molecular relationship of SIRT1 to type 2 diabetes and cancer. A variety of SIRT1 ligands with binding specificity have already been reported. Specific SIRT1 inhibitors include tenovins^7^ and EX-527^8^, while SIRT1 activators include SRT1720, SRT2183, and SRT1460, although they might activate SIRT1 indirectly^9^. Further SIRT1 ligands still need to be discovered from natural products.

High-throughput screening (HTS) has been used to develop novel SIRT1 inhibitors^10^,^11^ and activators^11^,^12^,^13^. It was estimated that about 60 million chemical structures are available for HTS, but only 1% of these structures have been screened for SIRT1 activators^13^,^14^. Cost-effective ligand-based virtual screening (VS) would be a good option for identifying potential compounds *in silico* before HTS^15^. Even if the experimental information for compounds is scanty, VS can still accelerate the identification and optimisation of candidate compounds^16^,^17^. In this ligand-based VS study, inductive logic programming (ILP) was used to develop molecular search patterns, and molecular docking was performed to estimate the binding affinities of potential SIRT1 ligands. ILP can consider specific characteristics of compounds and human-generated rules as background knowledge to outperform traditional approaches^18^.

The objective of the present study was to construct quantitative structure–activity relationship (QSAR) models^19^ of SIRT1 ligands for VS^15^ of 1 444 880 chemical structures collected from two major active compound databases, i.e. Traditional Chinese Medicines@Taiwan Database^20^ and Traditional Chinese Medicine Integrated Database^21^. The molecular search results were validated by molecular docking using AutoDock Vina software^22^.

## Results and Discussion

### Selection and characteristics of studies

**Figure 2:**
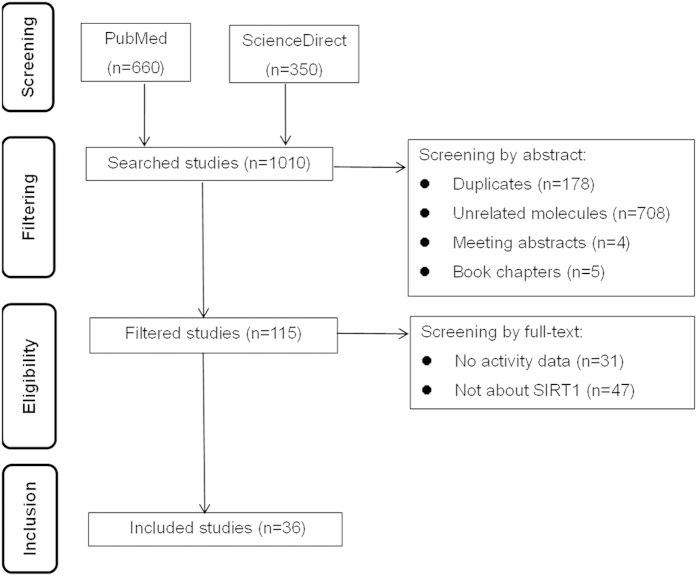
Flow diagram of the literature search and study selection.

A total of 1010 studies were retrieved from PubMed and ScienceDirect. After excluding 178 duplicates, the abstracts and full texts of the remaining 832 studies were screened and 36 eligible studies^7^,^10^,^11^,^12^,^23^,^24^,^25^,^26^,^27^,^28^,^29^,^30^,^31^,^32^,^33^,^34^,^35^,^36^,^37^,^38^,^39^,^40^,^41^,^42^,^43^,^44^,^45^,^46^,^47^,^48^,^49^,^50^,^51^,^52^,^53^,^54^. were included according to the study selection criteria. The flow diagram of the study selection is shown in Fig. 2.

As shown in Table 1, the eligible studies were published in the years between 2005 and 2013. There were 33 studies on SIRT1 inhibitors, two studies on SIRT1 activators, and one study on both activators and inhibitors. The three studies^12^,^19^,^37^ on SIRT1 activators employed HTS, which seemed to be the available approach in practice to screen for potential SIRT1 activators.

### Selection of ligands for modelling

A total of 482 compounds were identified from the 36 eligible studies. After removing 74 duplicates, the remaining 408 compounds were identified to be 354 inhibitors (Table S1) and 54 activators (Table S2). Three of the 54 activators lacked bioactivity data (i.e. EC_50_ and MA), and were not used for machine learning in activator model construction. According to PubChem^55^, a compound with an inhibitory effect had an IC_50_ below 50. Therefore, the 354 inhibitors were classified into three groups: 169 compounds were not significantly inhibitory (IC_50_ > 50, i.e. outcome = “unspecified” in Table S1); 179 were inhibitory (IC_50_ < 50, i.e. outcome = “active” in Table S1); and 6 had inconsistent outcomes in different studies (IC_50_ ≥ 50 in some studies and IC_50_ < 50 in other studies). Two compounds (SI27 and SI111) from the 169 compounds with implausible IC_50_ and six compounds with inconsistent outcomes were excluded from machine learning. According to PubChem^55^, 96 of the 179 inhibitor compounds were known to be inhibitors of both SIRT1 and SIRT2, possibly targeting the same catalytic core structure^56^.

### Model generation

*Activator model*. An activator model was built from 54 activators by setting “rank low” and EC_50_ < 2.15 as the cut-off criteria. As the area under the receiver-operating characteristic (ROC) curve (AUC) was only 0.67 and only a limited number of activators from three studies were available for modelling, the generated model would not be unbiased. Therefore, we did not use the activator model for further screening and focused on screening for inhibitors.

**Figure 3:**
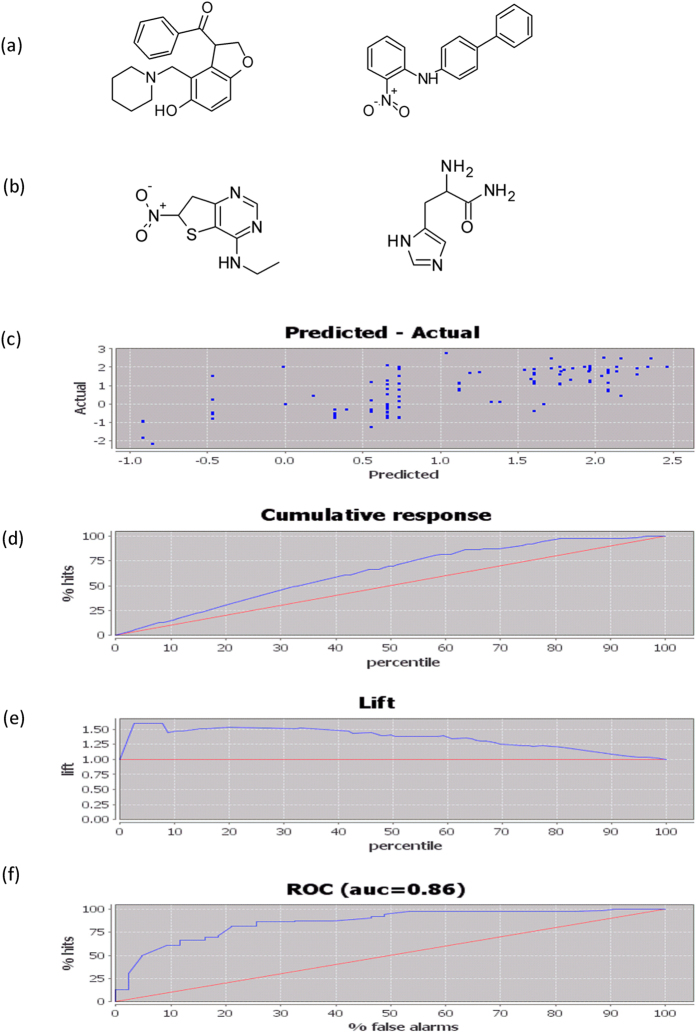
Reference structures and performance of the inhibitor model. (**a**) Reference structures of inhibitors with high IC_50_ values. (**b**) Reference structures of inhibitors with low IC_50_ values. (**c**) Predicted–actual scatter diagram of the inhibitor model. (**d**) Cumulative response curve of the inhibitor model, showing the percentage of hits (y-axis) within the first n percent of data (x-axis). (**e**) Lift curve of the inhibitor model, showing observation from the first top n percent of data about how many times the model outperformed a random model (y-axis). (**f**) ROC curve of the inhibitor model, showing the percentage of non-hits (x-axis: false alarms) to obtain a particular percentage of hits.

*Inhibitor model*. A total of 346 inhibitors were used to construct the inhibitor model, for which we performed a three-fold cross-validation. N1 inhibitors were randomly selected from the 346 inhibitors to construct a learning dataset, and the remaining N2 inhibitors were used as a testing dataset. The generated inhibitor model (hypotheses) suggested that inhibitors with specific structures containing two benzene rings and amine may have high IC_50_ values (P = 1.32 × 10^**−**4^, Table S3) and that inhibitors with specific structures containing amine, amide, and hetero-aromatic five-membered rings may have low IC_50_ values (P = 1.16 × 10^**−**4^, Table S3). The reference compound structures for these two hypotheses are shown in Fig. 3a,b, respectively.

Under the cut-off criteria of “rank low” and IC_50_ < 50, the AUC, the root mean square error (RMSE), Pearson correlation coefficient (r), and Spearman’s rank correlation coefficient (rho) of the model were satisfactory at 0.86, 0.79, 0.75, and 0.74, respectively. The scatter diagram with predicted values and experimental values is shown in Fig. 3c. The cumulative response curve (Fig. 3d) and lift curve (Fig. 3e) of the inhibitor model showed better performance of the model than stochastic ranking. The ROC curve (Fig. 3f) indicated that the model was accurate in identifying inhibitors. Therefore, we applied this model to screen natural product compounds for potential inhibitors of SIRT1.

*Differential model*. The differential model to distinguish between activators and inhibitors was built from bioactive ligands including 54 activators and 179 inhibitors (reference to Tables S1 and S2). The model indicated that inhibitor compounds contain thioamide (P = 1.84 × 10^**−**3^, Table S4) and activator compounds contain nitrogen heterocyclic five-membered ring, benzene ring, and amide (P = 1.21 × 10^**−**9^, Table S4). The confusion matrix of the differential model is shown in Table S5. Table S5 provides mis-classification details, showing that the model satisfactorily distinguished between inhibitors and activators. The reference compound structure with activation activity is shown in Figure S1. The differential model did not find a reference compound structure for inhibitors. This is probably based on the fact that the available activators were too few in number and too similar in structure as counter-examples to help generalise the inhibitor structures through inductive reasoning.

**Figure 4:**
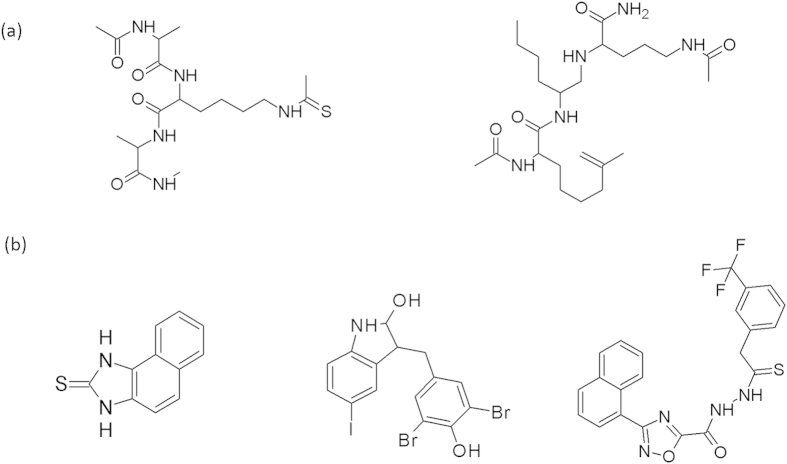
Reference structures of the inhibitor binding model. (**a**) Reference structures of inhibitors with high binding energy. (**b**) Reference structures of inhibitors with low binding energy.

*Inhibitor binding model*. To survey the binding energy profiles of the inhibitors, we conducted molecular docking on 178 known inhibitor compounds to estimate the inhibitor binding energy (Table S6). The binding energy between NAD^+^ and SIRT1, i.e. −7.1 kcal/mol, was regarded as a reference value. The binding energy information together with other required chemical information was fed in the DMax Chemistry Assistant (DCA) software^18^ to generate the inhibitor binding model. The generated inhibitor model suggested that compounds containing methyl, amide (thioamides, etc.), and aliphatic chains would have high binding energy (P = 0.01, Table S7) and that compounds containing two benzene rings, a general (hierarchy of moieties definition) functional group, and rings would have low binding energy (P = 6.52 × 10^**−**4^, Table S7). The reference compound structures of these two hypotheses are shown in Fig. 4a,b, respectively.

With the cut-off criteria of “rank low” and “binding energy <−6.0 kcal/mol”, the AUC, RMSE, r, and rho of this model were 0.9, 0.62, 0.68, and 0.67, respectively. The predicted *vs*. actual curve for the model is shown in Figure S2a. Sorting quality curves are shown in Figures S2b–S2d. All of these results showed a fair performance of the model.

*Inhibitor affinity model*. An inhibitor affinity model was generated using categorical variables and an inhibitor binding model was generated using numerical variables to investigate whether these two models were well-matched and whether the two methods were feasible for achieving the same goal, which was to find potential high-affinity compounds. Among the 178 known inhibitors shown in Table S7, only 23 compounds had binding energy lower than or equal to the reference value, which was too low to form a good model. We examined several cut-off values to divide the inhibitors into high-affinity and low-affinity compounds. Finally, −6.0 kcal/mol was regarded as the cut-off value, under which criterion high-affinity inhibitors (labelled with ‘a’) had binding energy <−6.0 kcal/mol and the others were low-affinity inhibitors (labelled with ‘b’). The affinity information together with other required chemical information was fed into the DCA software^18^ to generate an inhibitor affinity model with the highest model precision (78.26%), lowest P-value (3.58 × 10^−4^), and largest ROC (0.87) among all the attempts. The generated inhibitor affinity model suggested that compounds containing a ring, two benzene rings, and a general functional group would have high affinity (6.93 × 10^−6^, Table S8). The reference structures are shown in Figure S3. The model also suggested that compounds containing methyl, general amide, and aliphatic chain might have low affinity (P = 2.82 × 10^−3^, Table S8). The confusion matrix of this model is shown in Table S9, which shows that the model could separate the inhibitors of high affinity from those of low affinity.

Potential inhibitors with high affinity were investigated by two inhibitor models: the inhibitor binding model and the inhibitor affinity model. Under the cut-off criteria of “rank low” and “binding energy <−6.0 kcal/mol”, the AUC of the inhibitor binding model was 0.9 (Figure S2d), indicating its high quality of prediction. As shown in Table S7, the credibility of assuming a structure with low binding energy (i.e. high affinity) was obviously higher than the credibility of assuming a structure with high binding energy (i.e. low affinity) in the model. Under the same cut-off criterion (−6.0 kcal/mol), the inhibitor affinity model performed well (AUC = 0.87) as the inhibitor binding energy model and their characteristics were extremely well-matched (Table 2). It appeared that both of these models could be effective approaches to find potent inhibitors with low binding energy (i.e. high affinity). Integrating the results of both the binding and affinity models, we found that some ligands did have low affinity for SIRT1, but a significant inhibitory effect. This finding supported a previous study showing that SIRT1 ligands were not in simple competition with the substrate, but in a mixed-type process^57^. These models (Table 2) mainly covered the low-affinity inhibitors, which are acceptable for database screening purposes.

The DCA software^18^ only considered two-dimensional (2D) molecular structures, and could thus ignore some compounds with different 2D molecular descriptors but similar three-dimensional (3D) structural features to the bioactive compounds, e.g. SI6^11^. The DCA software^18^ would also ignore the chirality of chemical compounds that may lead to differences in biological activity.

As detailed bioactivity information was not available, the inhibitor models were actually generated from multiple categories of inhibitors, which might have different action mechanisms. Further studies should be conducted as soon as the detailed bioactivity information is available.

### Ligand-based virtual screening

**Figure 5:**
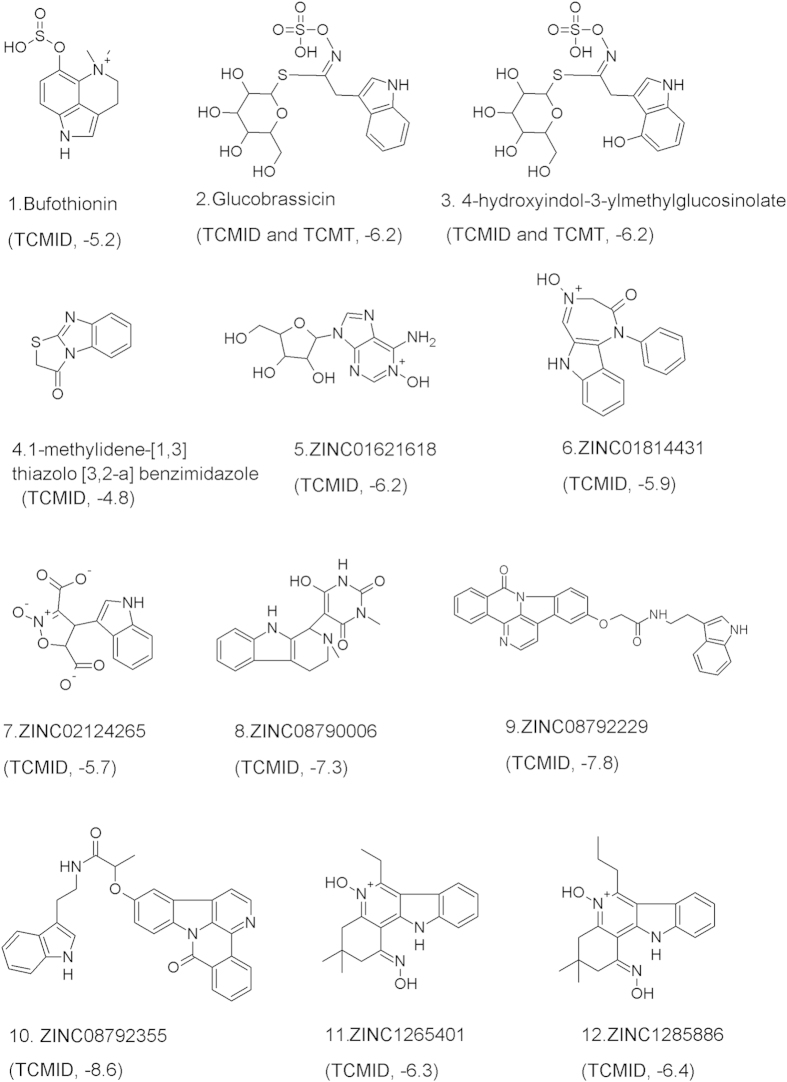
Structures (source, binding energy) of the 12 potential inhibitors identified by virtual screening. TCMT: Traditional Chinese Medicines@Taiwan^20^; TCMID: Traditional Chinese Medicine Integrated Database^21^.

We performed database screening with the inhibitor models and molecular docking to estimate the affinity of potential inhibitors. For the database screening, we downloaded chemical information from the Traditional Chinese Medicines@Taiwan database^20^ and Traditional Chinese Medicine Integrated Database^21^ and reconciled their format differences. We only used the inhibitor models for database screening because of their better predictive performance, as found in the model generation process. Twelve inhibitor candidates were identified by database screening based on the inhibitor models. The molecular features and binding energies of the candidates were further estimated by molecular docking (Fig. 5). Among the 12 compounds, the binding energies of three compounds (ZINC08790006, ZINC08792229, and ZINC08792355) were less than −7.1 kcal/mol and within the high-affinity range. The binding energies of the other nine compounds were between −4.8 and −6.4 kcal/mol and within the low-affinity range. The structures of the high-affinity compounds contained amide, amine, and hetero-aromatic five-membered ring, in accordance with the generated inhibitor models. The basic properties of these compounds (Table 3) obeyed Lipinski’s Rule of Five^58^, except for the large molecular weight and LogP3 value of compound ZINC08792355. As the numbers of H-bond donors and H-bond acceptors were less than 5 and the rotatable bond count was not more than 6, compound ZINC08792355 would have poorer absorption or permeability than the other two candidate compounds. This information would be useful to prioritise the candidate compounds for further laboratory testing.

### Significance of the study

This study is the first to apply an inductive learning technique to generate molecular models of SIRT1 inhibitors. Use of the molecular models in database screening before molecular docking reduced the time and cost of screening through molecular docking alone. The whole process of the ligand-based VS required hours rather than days. This study is also the first to apply ligand-based VS to screen for active compounds in natural products, particularly traditional Chinese medicines.

This study successfully demonstrated a use of the ILP approach to ligand-based virtual screening, based on machine learning from the structures of experimentally confirmed inhibitors (positive examples) and activators (negative examples), as well as optional background knowledge about the desirable targets. Although this study covered only a specific kind of chemical compounds (i.e. SIRT1 inhibitors), this approach can be generalized and applied to the virtual screening for other chemicals that require rich knowledge representations and automated reasoning.

## Methods

### Literature search

Two reviewers independently conducted database searches in PubMed and ScienceDirect. The search strategy was: (TITLE-ABSTR-KEY (sirt1) or TITLE-ABSTR-KEY (sirtuin 1)) and (TITLE-ABSTR-KEY (activator) or TITLE-ABSTR-KEY (inhibitor) or TITLE-ABSTR-KEY (agonist) or TITLE-ABSTR-KEY (antagonist) or TITLE-ABSTR-KEY (binder) or TITLE-ABSTR-KEY (ligand)). The publication language was English. The last search date was 20 February 2014.

Eligible articles were scientific experiment reports on biological activities of SIRT1 ligands with information on IC_50_, EC_50_, and maximum activation (MA). Articles were excluded if they were: (i) not original research articles; (ii) lacking in biological activity data; or (iii) lacking in chemical structures of the ligands.

Information was extracted from each eligible study, including first author, publication year, bioassay methods, substrates used in bioassays, and chemical information of discovered ligands.

### Data preparation

2D chemical structures of the ligands reported in eligible studies were re-sketched with ChemSketch software^59^. A compound search for chemical information from PubChem^55^ was performed with the search function of ChemSketch^59^. Canonical simplified molecular input line entry specification (SMILES), ID code, and all bioactivity information of the compounds were compiled in datasets and saved in sdf format (MDL MOL format) by OpenBabel 2.3.2 software^60^ with the settings of “add hydrogen to polar atoms only”, “canonicalize the atom order”, “generate 2D coordinates”, and “use wedge and hash bonds from input”. The chirality of the ligands was disregarded in the present study. Duplicate records were removed so that each record was unique. SIRT1 activators were indicated by IDs with prefix “SA” and SIRT1 inhibitors were indicated by IDs with prefix “SI”. The files containing the compound information in appropriate format were used in subsequent QSAR modelling.

### QSAR modelling by inductive learning

To relate the common structural compound features of the SIRT1 ligands to their bioactivities in QSAR modelling^13^ by machine learning, this study employed ILP-based DCA software^18^,^61^, ILP was applied in the modelling, including hierarchical hypotheses derivation, validation and deployment steps^18^. As an artificial intelligence method, ILP represents a particular model internally as formal logics that would facilitate inductive reasoning among data (examples or facts), background knowledge (facts or rules), and hypotheses (rules). ILP generates a more generic hypothesis to cover, subsume, or entail the given data and background knowledge. For non-computer scientists as its users, the DCA software employs logic formulae as its internal representations for inductive reasoning. The DCA software translates the input (data/examples and background knowledge) into or the output (hypotheses) from the internal logic formulae for more friendly interactions with the users. The workflow of DCA software is shown in Figure S4, and the models (in both English text and chemical structures) displayed by the DCA software is shown in Figure S5. For efficiency, the DCA software also incorporates simpler algorithms such as SVM and statistical regressions for specific classification tasks that do not require logical reasoning^62^. The parameters for these additional algorithms were automatically set by the DCA software. In the individual hypotheses generation step, DCA^18^ took advantage of the ILP capability for incorporating background knowledge. The background knowledge in DCA^18^ was divided into four parts: electron flow; element (e.g. carbon, nitrogen); moiety (functional groups and rings); and substructure relationship (e.g. connected, fused, linked, and position on ring). DCA^18^ could add vertices for all moieties to an atom-bond graph, connect the vertices and molecular structure elements with edges labelled by the substructure relationship, and then find correlation rules between the molecular structure information represented by the atom-bond graph and its experimental biological activity^63^. Users can set parameters for optimisation between model quality and run time. In this research, we explored with different the parameter settings and finally optimized the parameters as shown in Table S10, which were chosen for good model quality for screening purpose within an acceptable short period of time. The last parameter “both high and low values” in Table S10 aimed to broaden the search space (beyond “low values only” or “high values only” settings) for the model information. We also find that minor changes around the chosen parameter settings would return similar results. Generally, the dataset input for hypotheses formation was divided into a training set and a test set based on their structure diversity and activity performed by the software. The hypotheses were generated using the training set by machine learning and automatically validated by the test set data. The hypothesis validation was performed to observe whether the hypothesis was capable of distinguishing between active and inactive ligands^64^, and the statistical significance (e.g. P-values) was determined. We considered the significance level to be high for values of P < 0.005. Multiple descriptors and curves were used to present the performance of the models. A good model was characterized by a large AUC, low RMSE, good r, and good rho. Predicted–actual scatter plots indicated the Pearson correlations between the predicted and actual target values, while rank correlation values showed the correlations between the predicted ranks and actual ranks. Curves shown in red represented the performance of a random model which acted as a non-biased baseline, while curves shown in blue indicated the performance of the predictive models in the present study. In cumulative response curves, if the blue line was above the red line, the predictive model outperformed the random model. Lift curves showed how many times the predictive model outperformed the random model. ROC curves took the AUC between the blue line and the red line (0 ≤ AUC ≤ 1) to show how much better the predictive model was compared with the random model.

In operations, compound information files containing the data sets were fed into the DCA^18^ software to generate QSAR models, for which five descriptive models were constructed: (i) activator model; (ii) inhibitor model; (iii) differential model (i.e. ligand model that distinguished between activators and inhibitors); (iv) inhibitor binding model that distinguished between inhibitors with high and low binding energies; and (v) inhibitor affinity model that distinguished between high-affinity and low-affinity inhibitors. The binding energy and affinity between each inhibitor and SIRT1 were determined by molecular docking (see section titled *Molecular Docking*).

### Ligand-based virtual screening

We performed VS using the “apply hypotheses” option in the DCA software^18^. The validated QSAR models were used to screen natural product compounds, for which data were downloaded from the two major databases (Traditional Chinese Medicines@Taiwan Database^20^ and Traditional Chinese Medicine Integrated Database^21^), converted, and saved in sdf format by OpenBabel software^60^. Duplicate and incomplete records were removed. Finally, candidates were screened with predicted values (e.g. logIC_50_) and then taken for further molecular docking (see section titled *Molecular Docking*).

### Molecular docking

**Figure 6:**
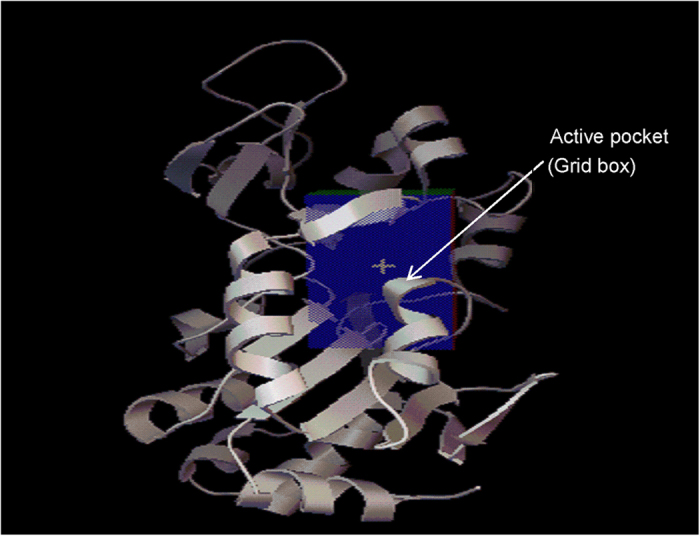
SIRT1 active pocket.

To investigate the intermolecular interactions between ligands and SIRT1, we performed semi-flexible docking using AutoDock Vina software^22^. 3D structural information for SIRT1 protein was obtained from the Protein Data Bank (PDB; ID: 4I5I). The information for the catalytic domain of SIRT1 (Fig. 6) and NAD^+^ protein-binding site was used for molecular docking as previously reported^65^. Hydrogen atoms were added to prepare the receptor file, for which the 3D structure was saved in pdbqt format by AutoDock Tools^66^. The ligand file was prepared in the same manner. The receptor and ligand files were then applied to docking in the AutoDock Vina software, which also estimated the binding energy and affinity^22^.

## Additional Information

**How to cite this article**: Sun, Y. *et al*. Ligand-based virtual screening and inductive learning for identification of SIRT1 inhibitors in natural products. *Sci. Rep*. **6**, 19312; doi: 10.1038/srep19312 (2016).

## Supplementary Material

Supplementary Information

## Figures and Tables

**Figure 1:**
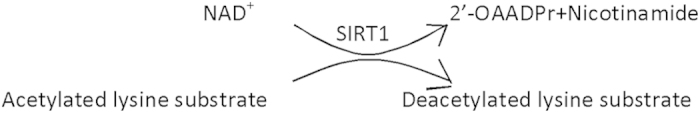
A reaction catalysed by SIRT1.

**Table 1:** Characteristics of eligible studies.

First author	Year	Bioassay	Substrate	Number	Target	Ligand type
Alvala	2012	fluorimetric assay	residues 379–382 of p53 (Arg-His-Lys-Lys (Ac))	8	SIRT1	inhibitor
Amagata	2012	fluorimetric assay	Arg-His-Lys-Lys (epsilon-acetyl)-AMC	2	SIRT1,SIRT2	inhibitor
Asaba	2008	fluorimetric assay	residues 379–382 of p53 (Arg-His-Lys-Lys (Ac))	24	SIRT1	inhibitor
Bemis	2009	unspecified	unspecified	30	SIRT1	activator
Disch	2013	mass spectrometry assay	Ac-RHKKAcW-NH2	37	SIRT1,SIRT2,SIRT3	inhibitor
Freitag	2011	fluorimetric assay	ZMAL	6	SIRT1,SIRT2,SIRT3	inhibitor
Hirsch	2011	HPLC	H2NHK-AcK-LM-COOH	3	SIRT1,SIRT2,SIRT3	inhibitor
Huber	2010	fluorimetric assay	ZMAL	2	SIRT1,SIRT2,SIRT3	inhibitor
Huhtiniemi	2010	fluorimetric assay	residues 379–382 of p53 (Arg-His-Lys-Lys (Ac))	14	SIRT1,SIRT2	inhibitor
Huhtiniemi	2011	fluorimetric assay	residues 379–382 of p53 (Arg-His-Lys-Lys (Ac))	20	SIRT1,SIRT2	inhibitor
Huhtiniemi	2008	Microplate filtration assay	residues 379–382 of p53 (Arg-His-Lys-Lys (Ac))	5	SIRT1,SIRT2	inhibitor
Kalle	2010	fluorimetric assay	residues 379–382 of p53 (Arg-His-Lys-Lys (Ac))	1	SIRT1	inhibitor
Kiviranta	2007	fluorimetric assay	residues 379–382 of p53 (Arg-His-Lys-Lys (Ac))	3	SIRT1,SIRT2	inhibitor
Kiviranta	2009	fluorimetric assay	residues 379–382 of p53 (Arg-His-Lys-Lys (Ac))	23	SIRT1,SIRT2	inhibitor
Mai	2005	fluorimetric assay	residues 379–382 of p53 (Arg-His-Lys-Lys (Ac))	4	Sir2,SIRT1,SIRT2	inhibitor
Mai	2009	fluorimetric assay	unspecified	2	SIRT1,SIRT2,SIRT3	activator, inhibitor
Manjulatha	2012	fluorimetric assay	residues 379–382 of p53 (Arg-His-Lys-Lys (Ac))	2	SIRT1	inhibitor
McCarthy	2012	fluorimetric assay	residues 379–382 of p53 (Arg-His-Lys-Lys (Ac))	22	SIRT1,SIRT2	inhibitor
Medda	2009	fluorimetric assay	residues 379–382 of p53 (Arg-His-Lys-Lys (Ac))	8	SIRT1,SIRT2	inhibitor
Napper	2005	fluorimetric assay	residues 379–382 of p53 (Arg-His-Lys-Lys (Ac))	24	SIRT1	inhibitor
Pasco	2010	fluorimetric assay	residues 379–382 of p53 (Arg-His-Lys-Lys (Ac))	12	SIRT1,SIRT2	inhibitor
Pesnot	2011	fluorimetric assay	ZMAL	1	SIRT1,SIRT2	inhibitor
Rotili	2011	fluorimetric assay	residues 379–382 of p53 (Arg-His-Lys-Lys (Ac))	6	SIRT1,SIRT2	inhibitor
Rotili	2012	fluorimetric assay	residues 379–382 of p53 (Arg-His-Lys-Lys (Ac))	14	SIRT1,SIRT2	inhibitor
Sanders	2009	fluorimetric assay	residues 379–382 of p53 (Arg-His-Lys-Lys (Ac))	14	Hst2,SIRT1	inhibitor
Suzuki	2009	fluorimetric assay	residues 379–382 of p53 (Arg-His-Lys-Lys (Ac))	2	SIRT1,SIRT2,SIRT3	inhibitor
Suzuki	2009	fluorimetric assay	residues 379–382 of p53 (Arg-His-Lys-Lys (Ac))	10	SIRT1	inhibitor
Suzuki	2006	fluorimetric assay	residues 379–382 of p53 (Arg-His-Lys-Lys (Ac))	10	SIRT1	inhibitor
Suzuki	2012	fluorimetric assay	residues 379–382 of p53 (Arg-His-Lys-Lys (Ac))	68	SIRT1,SIRT2	inhibitor
Tavares	2009	fluorimetric assay	unspecified	12	Sir2,SIRT1	inhibitor
Trapp	2006	fluorimetric assay/scintillation	ZMAL	4	SIRT1,SIRT2	inhibitor
Trapp	2007	fluorimetric assay	ZMAL	19	SIRT1,SIRT2	inhibitor
Uciechowska	2008	fluorimetric assay	ZMAL	7	SIRT1,SIRT2	inhibitor
Vu	2009	Mass Spectrometry Assay	derived from the sequence of p53	25	SIRT1	activator
Wu	2013	fluorimetric assay	Ac-RHKKAc-AMC	22	SIRT1	inhibitor
Zhang	2009	fluorimetric assay	unspecified	1	SIRT1,SIRT2, SIRT3	inhibitor

**Table 2:** Summary comparison of three models.

Model	Parameter	Structural characteristics
Inhibitor model	inhibitory effect	amides, amines, hetero-aromatic five-membered rings
Inhibitor binding model	high binding energy	methyl, general amide groups, aliphatic chains
	low binding energy	two benzene rings, a general functional group, rings
Inhibitor affinity model	high affinity	a ring, two benzene rings and a general functional group
	low affinity	methyl, general amide and aliphatic chain

**Table 3:** Properties of three potential SIRT1 inhibitors.

Properties	ZINC08790006	ZINC08792229	ZINC08792355
Molecular Weight (g/mol)	326.34982	486.52068	500.54726
Molecular Formula	C17H18N4O3	C30H22N4O3	C31H24N4O3
XLogP3-AA	0.8	4.9	5.3
H-Bond Donor	3	2	2
H-Bond Acceptor	4	4	4
Rotatable Bond Count	1	6	6
Topological Polar Surface Area	88.7	89	89
Heavy Atom Count	24	37	38
Formal Charge	0	0	0
Complexity	65	868	898
