# Supplementary material for: Ligand-based virtual screening and inductive learning for identification of SIRT1 inhibitors in natural products
Source: Sci Rep. 2016 Jan 25;6:19312. doi: 10.1038/srep19312 (PMC4726279; doi:10.1038/srep19312)
Supplement: Supplementary Information [file srep19312-s1.pdf]

Ligand-based virtual screening and inductive learning for identification of SIRT1 inhibitors in natural products

Yunan Sun[1]*, Hui Zhou[1]*, Hongmei Zhu[1] and Siu-wai Leung[1,2,]**

[1]State Key Laboratory of Quality Research in Chinese Medicine, Institute of Chinese Medical Sciences, University of Macau, Macao, China
[2]School of Informatics, University of Edinburgh, Edinburgh EH8 9AB, United Kingdom
* Co-first authors
** Corresponding author

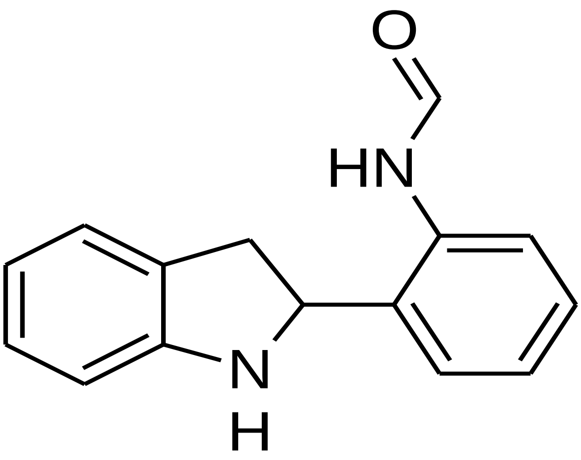

Figure S1 A reference structure of activators in the differential model.

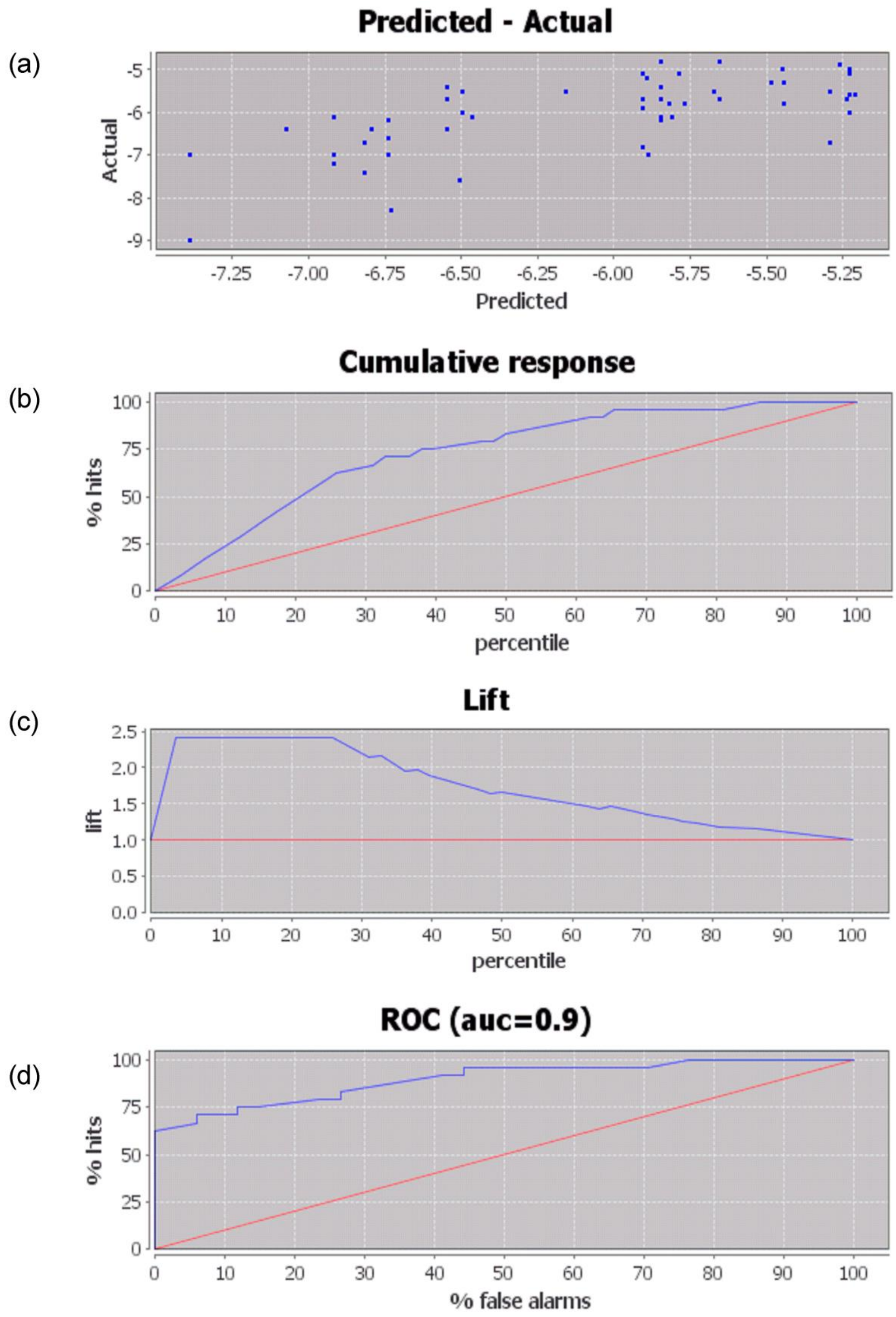

Figure S2 Performance of the inhibitor binding model. (a) Predicted-actual scatter diagram of the inhibitor binding model. (b) Cumulative response curve of the inhibitor binding model. (c) Lift curve of the inhibitor binding model. (d)

ROC of the inhibitor binding model.

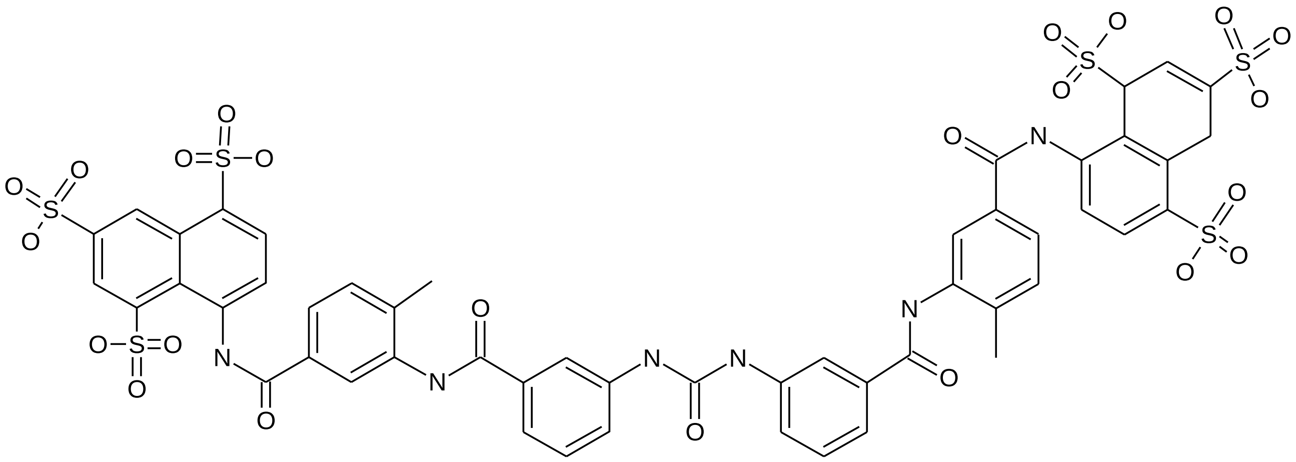

Figure S3 A reference structure of the inhibitors with high affinity.

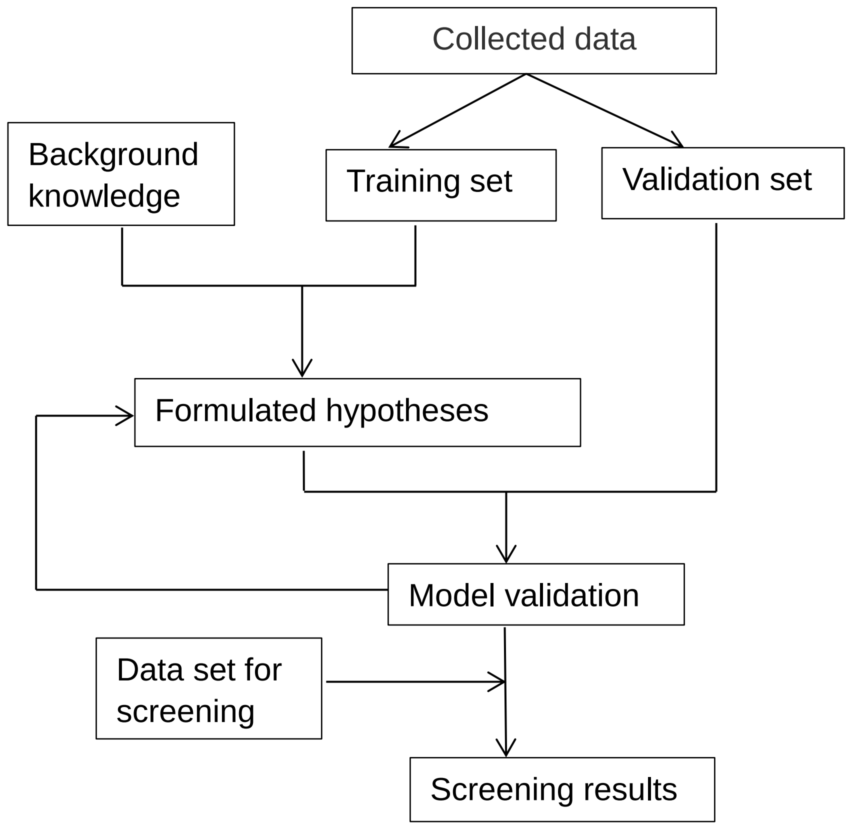

Figure S4 Workflow of the DCA software.

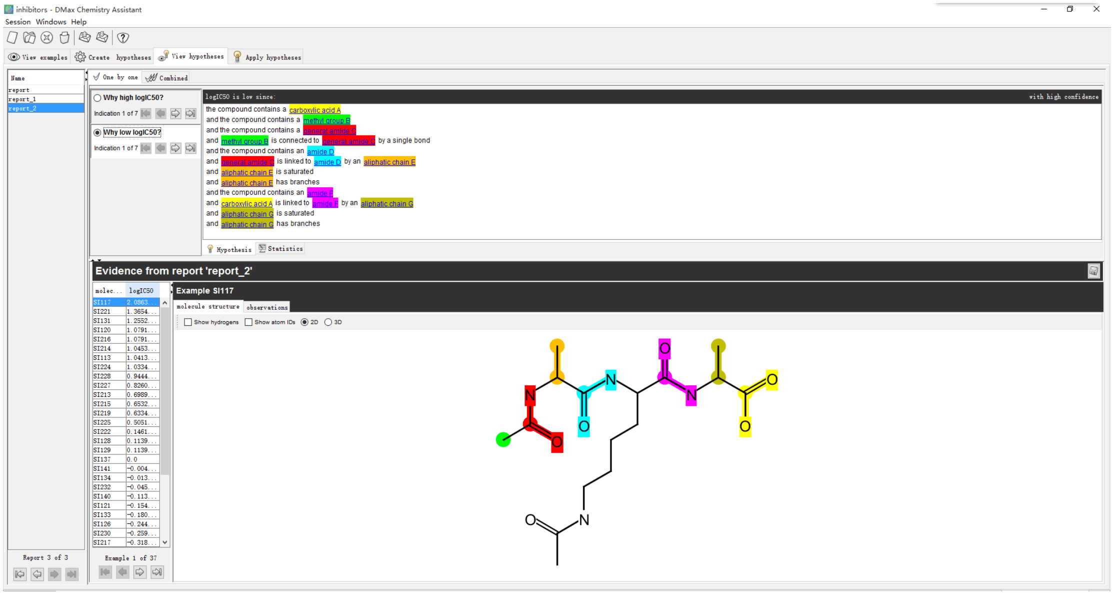

Figure S5 A hypothesis generated from Dmax Chemistry Assistant (DCA). The generated hypothesis is represented by both English text and chemical structure. Corresponding moieties are highlighted by the same colors in the English text and chemical structure.

Table S1 List of SIRT1 inhibitors collected from eligible studies

ID	CID	IC_{50} (μm)	Outcome
SI1	5083	25, 5.1, 3.5	active
SI2	5361	0.6, 2.8, 2.6, 0.3	active
SI3	648461	80	unspecified
SI4	5269	500, 96.2	unspecified
SI5	936	250, 90.4, 100, 520	unspecified
SI6	3612016	0.18, 0.12, 0.65, 0.06, 23	active
SI7	5717148	120, 123, 68, 40.1, 131, 123.3, 55, 67	active/unspecified
SI8	443592	600	unspecified
SI9	3246390	57.9, 56, 40.7, 600	active/unspecified
SI10	24772043	100	unspecified
SI11	46226668	0.64	active
SI12	46226669	6	active
SI13	46226675	34	active
SI14	24168039	570	unspecified
SI15	5811220	73	unspecified
SI16	44427280	52	unspecified
SI17	5113032	0.1, 0.28, 1.29, 0.16, 0.26	active
SI18	2396	50	active/unspecified
SI19	2400	50	active/unspecified
SI20	5331125	50	active/unspecified
SI21	1104317	9.6	active
SI22	703333	29.3	active
SI23	11126879	21.4	active
SI24	56659567	7.7	active
SI25	56676724	100	unspecified
SI26	42626405	43	active
SI27	71456062	3700	unspecified
SI28	11696870	59	unspecified
SI29	11509437	13, 48.1	active
SI30	2130404	30, 38, 50	active/unspecified
SI31	44565975	89.8	unspecified
SI32	46179785	44.1	active
SI33	46179786	40.3	active
SI34	46231289	8.4	active
SI35	1403654	5.3	active
SI36	56669934	12.4	active
SI37	53257841	17.5	active
SI38	16805603	112	unspecified
SI39	658354	300	unspecified
SI40	1211361	145	unspecified
SI41	787462	300	unspecified

ID	CID	IC_{50} (μm)	Outcome
SI42	746633	256	unspecified
SI43	626866	300	unspecified
SI44	71717360	31.2	active
SI45	71717361	88	unspecified
SI46	687118	2.8	active
SI47	645092	300	unspecified
SI48	71717955	41.7	active
SI49	867287	300	unspecified
SI50	1207797	198	unspecified
SI51	1068167	300	unspecified
SI52	71718571	71.4	unspecified
SI53	900927	240	unspecified
SI54	878744	300	unspecified
SI55	71719783	45.1	active
SI56	1736412	300	unspecified
SI57	877268	243	unspecified
SI58	1208250	300	unspecified
SI59	787460	300	unspecified
SI60	71453621	1	active
SI61	60150584	10.13	active
SI62	70685393	40.06	active
SI63	70685403	25.02	active
SI64	60150585	43.29	active
SI65	70691733	45.97	active
SI66	60150563	11.08	active
SI67	60150564	43.14	active
SI68	661302	0.2, 2.5	active
SI69	4262314	1.47, 3.3	active
SI70	2859649	100	unspecified
SI71	2915792	100	unspecified
SI72	609412	100	unspecified
SI73	3148372	100	unspecified
SI74	11694306	15	active
SI75	11687587	77.6	unspecified
SI76	11536113	34.5	active
SI77	11565551	2.44	active
SI78	2858149	100	unspecified
SI79	2848181	100	unspecified
SI80	2831741	100	unspecified
SI81	11854565	13	active
SI82	44405122	100	unspecified
SI83	11701546	18	active
SI84	11522166	100	unspecified

ID	CID	IC_{50} (μm)	Outcome
SI85	11652227	79.9	unspecified
SI86	11708474	0.41	active
SI87	11708252	2.67	active
SI88	45483031	2.7, 9.9	active
SI89	49847185	90	unspecified
SI90	57391607	35.8	active
SI91	49847195	42.7	active
SI92	57392690	90	unspecified
SI93	49847269	36.7	active
SI94	49847191	90	unspecified
SI95	49847187	90	unspecified
SI96	57395072	16.1	active
SI97	57395074	60	unspecified
SI98	49847119	46.3	active
SI99	57398537	70.9	unspecified
SI100	49871498	37.5	active
SI101	57398601	24.7	active
SI102	49847038	90	unspecified
SI103	49847117	39.9	active
SI104	49806787	18.3	active
SI105	57400269	60.2	unspecified
SI106	49847271	90	unspecified
SI107	57345649	21.7	active
SI108	57403130	17.9	active
SI109	49847189	90	unspecified
SI110	49847123	90	unspecified
SI111	52941053	1767	unspecified
SI112	52941276	0.37	active
SI113	52942684	11	active
SI114	46919126	1.8	active
SI115	46919223	223	unspecified
SI116	52943833	46	active
SI117	52944947	122	unspecified
SI118	52946220	40	active
SI119	52947355	0.24	active
SI120	52947577	12	active
SI121	52947608	0.7	active
SI122	52947718	6.1	active
SI123	52948629	268	unspecified
SI124	52949842	88	unspecified
SI125	44572666	0.31, 0.33	active
SI126	44572702	0.57	active
SI127	54757795	0.4	active

ID	CID	IC_{50} (μm)	Outcome
SI128	54669819	1.3	active
SI129	54757895	1.3	active
SI130	54669816	1.6	active
SI131	54757893	18	active
SI132	54757796	1	active
SI133	54757797	0.66	active
SI134	54669818	0.97	active
SI135	54757990	7.4	active
SI136	54757989	23	active
SI137	54757896	1	active
SI138	54757991	5.1	active
SI139	54757799	51	unspecified
SI140	54757892	0.77	active
SI141	54757894	0.99	active
SI142	54757800	0.37	active
SI143	42617956	13	active
SI144	96455	17, 56	active
SI145	45486324	40, 40.6	active
SI146	45267617	89	unspecified
SI147	45268494	68	unspecified
SI148	45269359	60	unspecified
SI149	45270178	58	unspecified
SI150	45270186	72	unspecified
SI151	2728632	78	unspecified
SI152	44596232	52	unspecified
SI153	44596233	58	unspecified
SI154	45272735	65	unspecified
SI155	4386	68	unspecified
SI156	45267626	100	unspecified
SI157	45267627	100	unspecified
SI158	43358927	100	unspecified
SI159	60167271	100	unspecified
SI160	60167272	100	unspecified
SI161	60167274	100	unspecified
SI162	60165377	100	unspecified
SI163	60165379	100	unspecified
SI164	71451254	100	unspecified
SI165	71451255	93	unspecified
SI166	71451256	100	unspecified
SI167	71451257	100	unspecified
SI168	60167126	100	unspecified
SI169	60167129	100	unspecified
SI170	60167270	100	unspecified

ID	CID	IC_{50} (μm)	Outcome
SI171	60165644	100	unspecified
SI172	60165647	100	unspecified
SI173	901929	56	unspecified
SI174	13505632	100	unspecified
SI175	70089831	100	unspecified
SI176	12476632	100	unspecified
SI177	60167277	100	unspecified
SI178	60167422	100	unspecified
SI179	60165646	100	unspecified
SI180	60165796	100	unspecified
SI181	60167273	100	unspecified
SI182	60165642	100	unspecified
SI183	60148504	100	unspecified
SI184	60165648	100	unspecified
SI185	60165794	100	unspecified
SI186	60165798	100	unspecified
SI187	15701548	100	unspecified
SI188	60165378	100	unspecified
SI189	60165380	210	unspecified
SI190	60165643	100	unspecified
SI191	60165645	100	unspecified
SI192	60165797	100	unspecified
SI193	60165799	100	unspecified
SI194	71458450	100	unspecified
SI195	3290567	100	unspecified
SI196	60167128	100	unspecified
SI197	60167276	100	unspecified
SI198	60167419	100	unspecified
SI199	60165381	300	unspecified
SI200	60165506	100	unspecified
SI201	60167423	100	unspecified
SI202	60165382	300	unspecified
SI203	56935695	100	unspecified
SI204	60165795	100	unspecified
SI205	67523832	75	unspecified
SI206	60167127	100	unspecified
SI207	60167418	100	unspecified
SI208	60167420	100	unspecified
SI209	60167421	100	unspecified
SI210	60167424	100	unspecified
SI211	60165383	300	unspecified
SI212	2255545	45.3	active
SI213	44572628	5	active

ID	CID	IC_{50} (µm)	Outcome
SI214	44572662	11.1	active
SI215	44572663	4.5	active
SI216	44572664	12	active
SI217	44572700	0.48	active
SI218	44572701	0.33	active
SI219	44572703	4.3	active
SI220	44572704	0.18	active
SI221	44572705	23.2	active
SI222	44572706	1.4	active
SI223	44572767	0.23	active
SI224	44572768	10.8	active
SI225	44572769	3.2	active
SI226	44572770	0.22	active
SI227	44572771	6.7	active
SI228	44572817	8.8	active
SI229	44572818	0.38	active
SI230	44572819	0.55	active
SI231	44572820	0.18	active
SI232	44572821	0.9	active
SI233	42617959	43	active
SI234	42617960	44.2	active
SI235	42617961	44.5	active
SI236	42617968	50	unspecified
SI237	42617969	38.3	active
SI238	42617970	90	unspecified
SI239	56674921	41.6	active
SI240	N/A	15	active
SI241	70676624	10.4	active
SI242	70676484	20.2	active
SI243	70676622	9.4	active
SI244	70676627	11.8	active
SI245	70676628	12.5	active
SI246	70676483	7	active
SI247	70676623	6.6	active
SI248	70676626	12	active
SI249	12761967	10.5	active
SI250	70676625	21.1	active
SI251	70676792	14.1	active
SI252	24949217	318	unspecified
SI253	24949372	168	unspecified
SI254	2743189	192	unspecified
SI255	24949066	325	unspecified
SI256	24949071	13	active

ID	CID	IC_{50} (μm)	Outcome
SI257	24822260	31	active
SI258	46871955	5.9	active
SI259	56664966	290	unspecified
SI260	53379498	89.5	unspecified
SI261	71654772	0.014	active
SI262	71654912	0.73	active
SI263	71654990	0.18	active
SI264	71654993	50	unspecified
SI265	71654775	15	active
SI266	71304814	0.015	active
SI267	71654987	0.47	active
SI268	71654988	0.059	active
SI269	71655060	0.0053	active
SI270	71304813	0.0036	active
SI271	71654773	0.0058	active
SI272	71655063	0.017	active
SI273	71654841	0.056	active
SI274	71654913	0.11	active
SI275	71654991	50	unspecified
SI276	71654909	0.042	active
SI277	71654771	0.0067	active
SI278	71655141	1.6	active
SI279	71654838	50	active
SI280	71654842	0.49	active
SI281	71654911	0.38	active
SI282	71654989	0.49	active
SI283	71654992	50	unspecified
SI284	71655062	0.031	active
SI285	71654774	0.11	active
SI286	71654770	50	unspecified
SI287	71654839	16	active
SI288	71654843	4.3	active
SI289	71304815	0.0043	active
SI290	71654914	0.053	active
SI291	71654840	1.6	active
SI292	71655061	0.067	active
SI293	71654910	0.12	active
SI294	8514	0.3	active
SI295	N/A	0.165	active
SI296	N/A	0.223	active
SI297	N/A	0.308	active
SI298	N/A	0.283	active
SI299	N/A	1.713	active

ID	CID	IC_{50} (μm)	Outcome
SI300	N/A	0.662	active
SI301	N/A	0.339	active
SI302	N/A	0.233	active
SI303	N/A	0.093	active
SI304	N/A	0.236	active
SI305	N/A	1.286	active
SI306	N/A	0.284	active
SI307	N/A	0.466	active
SI308	N/A	0.430	active
SI309	N/A	0.581	active
SI310	N/A	0.525	active
SI311	N/A	32.495	active
SI312	N/A	300	unspecified
SI313	N/A	300	unspecified
SI314	N/A	300	unspecified
SI315	N/A	300	unspecified
SI316	N/A	300	unspecified
SI317	N/A	300	unspecified
SI318	N/A	300	unspecified
SI319	N/A	35	active
SI320	N/A	3.9	active
SI321	N/A	62	unspecified
SI322	N/A	47	active
SI323	N/A	73	unspecified
SI324	N/A	300	unspecified
SI325	N/A	14	active
SI326	N/A	15	active
SI327	N/A	96	unspecified
SI328	N/A	300	unspecified
SI329	N/A	300	unspecified
SI330	N/A	300	unspecified
SI331	N/A	300	unspecified
SI332	N/A	88	unspecified
SI333	N/A	56	unspecified
SI334	N/A	300	unspecified
SI335	N/A	300	unspecified
SI336	5397113	12.9	active
SI337	694858	13.2	active
SI338	3311	47.90	active
SI339	3610426	50.50	unspecified
SI340	5380241	5.90	active
SI341	N/A	61.60	unspecified
SI342	694859	12.40	active

ID	CID	IC_{50} (μm)	Outcome
SI343	N/A	73.10	unspecified
SI344	N/A	82.20	unspecified
SI345	N/A	93.50	unspecified
SI346	N/A	127.50	unspecified
SI347	N/A	116.50	unspecified
SI348	N/A	97.40	unspecified
SI349	N/A	113.80	unspecified
SI350	N/A	94.70	unspecified
SI351	N/A	94.80	unspecified
SI352	N/A	102.20	unspecified
SI353	N/A	43.10	active
SI354	N/A	182.80	unspecified

Note: CID, compound number in PubChem; IC_{50}, concentration of compounds required to inhibit the SIRT1 activity by 50%; ID, compound number in the data set; N/A, not available; Outcome, inhibitory effect of compounds.

Table S2 List of SIRT1 activators collected from eligible studies

ID	CID	EC_{50} (μm)	MA (%)	Outcome
SA1	1114244	6	240	active
SA2	44591883	25	207	active
SA3	44591884	1.8	330	active
SA4	44591924	0.7	240	active
SA5	44591925	1.1	200	active
SA6	44591926	1.1	240	active
SA7	44591927	0.5	220	active
SA8	44591509	0.9	170	active
SA9	44591510	4.4	209	active
SA10	4274187	1.7	180	active
SA11	17497689	0.4	595	active
SA12	44591533	0.3	253	active
SA13	44591534	0.5	314	active
SA14	44138107	0.4	820	active
SA15	44591923	4.1	250	active
SA16	44591557	0.9	273	active
SA17	44591558	0.7	230	active
SA18	24769779	0.5	270	active
SA19	44591559	0.5	232	active
SA20	44591560	1.4	324	active
SA21	44591581	2.3	150	active
SA22	44591582	1.6	180	active
SA23	1104081	11	388	active
SA24	1298854	7.5	380	active
SA25	25232520	2.5	272	active
SA26	25231982	4.8	295	active
SA27	25231983	1.9	270	active
SA28	25232898	3.2	257	active
SA29	24180124	1.8	271	active
SA30	25232161	130	383	active
SA31	25233062	14	130	active
SA32	25232162	25	322	active
SA33	25233063	4.5	406	active
SA34	25232163	31	197	active
SA35	25233064	3.7	928	active
SA36	25232164	2.1	662	active
SA37	25232165	6.2	619	active
SA38	25233065	4.9	332	active
SA39	25232166	0.9	665	active
SA40	25233238	0.51	808	active
SA41	25232345	0.99	440	active
SA42	24180125	0.16	781	active

ID	CID	EC_{50} (μm)	MA (%)	Outcome
SA43	25233239	0.68	301	active
SA44	25232346	45	208	active
SA45	25232347	0.61	381	active
SA46	25233240	6.9	380	active
SA47	25232348	24	253	active
SA48	25232349	1.7	270	active
SA49	N/A	0.4	305	active
SA50	445154	46.2	201	active
SA51	24180126	0.36	296	active
SA52	N/A	N/A	N/A	active
SA53	10620479	N/A	N/A	active
SA54	16738819	N/A	N/A	active

Note: CID, compound number in PubChem; EC_{50}, concentration of compounds required to increase the SIRT1 activity by 50%; ID, compound number in the data set; MA, the percentage maximum activation achieved at the highest doses of compound tested; N/A, not available; Outcome, active effect of compounds.

Table S3 Statistical hypothesis testing of the inhibitor structures on IC_{50}.

Testing data set	Population[a]	Sub-population with high IC_{50}[b]	Sub-population with low IC_{50}[b]
Size	115[c]	34[d] (29.57%)	14[d] (12.17%)
Average	0.98[e]	1.81[f] (+0.83)[g]	-0.64[f] (-1.62)[g]

Note: [a] whole validation set; [b] subset of compounds covered by the hypothesis; [c] size of the whole validation set; [d] size of the subset; [e] average IC_{50} of compounds in the whole validation set; [f] average IC_{50} of compounds in the subset; [g] difference in average IC_{50} between the subset and the whole validation set.

Table S4 Statistical hypothesis testing of the inhibitor or activator structures in the differential model.

Testing data set	Population[a]	Sub-population of inhibitors[b]	Sub-population of activators[b]
Size	78[c]	24[d] (30.77%)	14[d] (17.95%)
Percent of inhibitors/activators	76.92[e]/23.08[f]	100%[g] (+23.08)[h]	100%[g] (+76.92)[h]

Note: [a] whole validation set; [b] subset of compounds covered by the hypothesis; [c] size of the whole validation set; [d] size of the subset; [e] percentage of compounds in the whole validation set of inhibitors; [f] percentage of compounds in the whole validation set of activators; [g] percentage of compounds in the subset of inhibitors or activators; [h] difference in percentage between the subset and the whole validation set.

Table S5 Confusion matrix of the differential model.

Prediction	Activator	Inhibitor	Total
Actual activator	61	0	61 (78.21%)
Actual inhibitor	2	15	17 (21.79%)
Total	63 (80.77%)	15 (19.23%)	78

Table S6 Binding energy between potential inhibitors and SIRT1.

ID	Binding energy (kcal/mol)	ID	Binding energy (kcal/mol)	ID	Binding energy (kcal/mol)
SI1	-7.70	SI122	-5.30	SI257	-7.00
SI2	-8.00	SI125	-5.50	SI258	-4.60
SI6	-5.80	SI126	-4.90	SI261	-4.80
SI11	-5.80	SI127	-5.00	SI262	-5.70
SI12	-5.90	SI128	-5.40	SI263	-5.60
SI13	-5.70	SI129	-5.60	SI265	-5.10
SI17	-6.10	SI130	-6.00	SI266	-5.50
SI21	-5.40	SI131	-6.00	SI267	-5.70
SI22	-5.50	SI132	-4.80	SI268	-5.90
SI23	-5.10	SI133	-5.10	SI269	-6.20
SI24	-5.20	SI134	-5.10	SI270	-5.70
SI26	-6.80	SI135	-5.70	SI271	-6.60
SI29	-7.40	SI136	-4.80	SI272	-5.40
SI32	-6.70	SI137	-5.70	SI273	-5.30
SI33	-6.80	SI138	-5.80	SI274	-5.90
SI34	-7.20	SI140	-4.80	SI276	-5.70
SI35	-5.90	SI141	-5.50	SI277	-5.70
SI36	-6.40	SI142	-5.20	SI278	-5.10
SI37	-6.70	SI143	-7.10	SI280	-5.40
SI44	-5.90	SI144	-5.90	SI281	-5.80
SI46	-6.10	SI145	-6.10	SI282	-5.80
SI48	-6.00	SI212	-6.20	SI284	-5.20
SI55	-5.50	SI213	-4.30	SI285	-5.80
SI60	-5.80	SI214	-5.10	SI287	-5.60
SI61	-7.60	SI215	-5.10	SI288	-5.50
SI62	-8.30	SI216	-5.10	SI289	-5.20
SI63	-8.50	SI217	-5.00	SI290	-5.70
SI64	-9.00	SI218	-6.70	SI291	-5.00
SI65	-8.60	SI219	-5.60	SI292	-6.10
SI66	-7.00	SI220	-4.40	SI293	-5.90
SI67	-7.00	SI221	-5.40	SI294	-6.90
SI68	-5.80	SI222	-5.20	SI295	-6.20
SI69	-5.80	SI223	-5.40	SI296	-7.10
SI74	-5.80	SI224	-4.50	SI297	-7.20
SI76	-5.80	SI225	-5.70	SI298	-7.20
SI77	-5.80	SI226	-5.00	SI299	-5.90
SI81	-6.00	SI227	-4.90	SI300	-5.40
SI83	-5.80	SI228	-5.30	SI301	-5.80
SI86	-6.00	SI229	-5.40	SI302	-6.60
SI87	-5.50	SI230	-5.50	SI303	-8.30
SI88	-6.10	SI231	-6.70	SI304	-6.40

ID	Binding energy (kcal/mol)	ID	Binding energy (kcal/mol)	ID	Binding energy (kcal/mol)
SI90	-6.60	SI232	-5.30	SI305	-7.00
SI91	-6.50	SI233	-7.30	SI306	-7.20
SI93	-5.00	SI234	-6.40	SI307	-7.20
SI96	-5.80	SI235	-7.40	SI308	-6.10
SI98	-5.60	SI237	-7.10	SI310	-7.20
SI100	-6.30	SI239	-6.70	SI311	-8.00
SI101	-5.50	SI240	-7.20	SI319	-6.40
SI103	-5.60	SI241	-6.60	SI320	-5.10
SI104	-6.50	SI242	-6.60	SI322	-6.40
SI107	-6.40	SI243	-6.20	SI325	-6.10
SI108	-5.90	SI244	-6.00	SI326	-6.50
SI112	-5.30	SI245	-5.70	SI336	-6.80
SI113	-5.30	SI246	-6.60	SI337	-6.50
SI114	-5.50	SI247	-6.60	SI338	-5.70
SI116	-5.40	SI248	-7.60	SI340	-6.20
SI118	-5.30	SI249	-6.40	SI342	-6.00
SI119	-4.90	SI250	-7.00	SI353	-5.40
SI120	-4.90	SI251	-5.40	NAD^+	-7.1
SI121	-5.20	SI256	-7.60		

Table S7 Statistical hypothesis testing of the inhibitor structures on binding energy

Testing data set	Population[a]	Sub-population with high binding energy[b]	Sub-population with low binding energy[b]
Size	58[c]	17[d] (29.31%)	19[d] (32.76%)
Average	-6.01[e]	-5.45[f] (+0.57)[g]	-6.82[f] (-0.8)[g]

Note: [a] whole validation set; [b] subset of compounds covered by the hypothesis; [c] size of the whole validation set; [d] size of the subset; [e] average binding energy of compounds in the whole validation set; [f] average binding energy of compounds in the subset; [g] difference in average binding energy between the subset and the whole validation set.

Table S8 Statistical hypothesis testing of the inhibitor structures on affinity

Testing data set	Population[a]	Sub-population with high affinity[b]	Sub-population with low sffinity[b]
Size	58[c]	19[d] (32.76%)	17[d] (29.31%)
Percentage of target	38.76[e]/61.24[f]	89.47%[g] (+50.71)[h]	94.12%[g] (32.88)[h]

Note: [a] whole validation set; [b] subset of compounds covered by the hypothesis; [c] size of the whole validation set; [d] size of the subset; [e] percentage of compounds in the whole validation set of inhibitors with high affinity; [f] percentage of compounds in the whole validation set of inhibitors with low affinity; [g] percentage of compounds in the subset of inhibitors with high or low affinity; [h] difference in percentage between the subset and the whole validation set.

Table S9 Confusion matrix of the inhibitor affinity model

Prediction	Inhibitor with high affinity	Inhibitor with low affinity	Total
Actual inhibitor with high affinity	18	6	24 (41.38%)
Actual inhibitor with low affinity	5	29	34 (58.62%)
Total	23 (39.66%)	35 (60.34%)	58

Table S10 Parameter settings for QSAR modeling in DCA software

	Maximal rule length	Field of view width	Allow overlap	Max specialization	Stop after	Option
Activator model	3	5	4	8	3	both high and low value
Inhibitor model	3	5	4	8	3	both high and low value
Differential model	3	5	4	8	3	both high and low value
Inhibitor binding model	5	6	7	10	6	both high and low value
Inhibitor affinity model	5	6	7	10	6	both high and low value
